# Late-Onset Wilson's Disease

**DOI:** 10.3389/fmed.2020.00026

**Published:** 2020-02-06

**Authors:** Miroslav Žigrai, Milan Vyskočil, Andrea Tóthová, Peter Vereš, Peter Bluska, Peter Valkovič

**Affiliations:** ^1^1st Department of Internal Medicine, Faculty of Medicine, University Hospital, Slovak Medical University, Bratislava, Slovakia; ^2^Department of Internal Medicine, Faculty of Medicine, University Hospital - St. Michael's Hospital, Slovak Medical University, Bratislava, Slovakia; ^3^Medicyt, s.r.o., Bratislava, Slovakia; ^4^Rádiológia, s.r.o., Bratislava, Slovakia; ^5^2nd Department of Neurology, Faculty of Medicine, University Hospital, Comenius University Bratislava, Bratislava, Slovakia

**Keywords:** Wilson's disease, late-onset, liver metastatic-like changes, copper, older age

## Abstract

Wilson's disease is a rare autosomal recessive disease, caused by impaired secretion of copper into bile due to a defective function of the ATPase 7B enzyme. Clinical manifestation is predominantly hepatic and neurological. Wilson's disease is traditionally considered a disease of children and young adults. It rarely manifests after 40 years of age. In our case report, we present a 67-year-old female in whom Wilson's disease manifested as tremors of the upper extremities and chin that were originally assessed as part of cerebral atherosclerosis and Parkinson's disease. Only the histological finding of liver steatofibrosis, performed due to suspected metastatic changes of the liver, led in the context of neurological symptoms to correct diagnosis and successful treatment.

## Introduction

Wilson's disease is a rare autosomal recessive disease, caused by impaired excretion into bile due to a defective function of the enzyme ATPase 7B in hepatocytes (1, 2). Accumulation of copper in various organs causes damage to them, with broad-ranging clinical symptoms dominated by signs of liver and brain damage (especially of the basal ganglia and cerebellum).

Wilson's disease is traditionally considered a disease of children and young adults. In our paper we present a patient with Wilson's disease, which manifested with neurological symptoms when she was in her 60s.

## Case Report

A 67-year-old female, with varicose veins in her legs, arterial hypertension, and type 2 diabetes mellitus controlled with diet, was evaluated by a neurologist in March 2012 for a 3-month-long development of upper extremities and chin tremors. The condition was firstly attributed to incipient cerebral atherosclerosis. Amantadine sulfate therapy was initiated. Later on in the differential diagnostics, essential tremor, and Parkinson's disease were suspected. A computed tomography (CT) of the brain revealed predominantly cortical atrophy of the brain frontally and bilaterally, with a marked atrophy of structures in the posterior cranial fossa.

For the next 2 years she was treated with the combination or alternation of vinpocetine, pramipexole, alprazolam, biperiden, and ropinirole, without significant reduction of her neurological symptoms.

Due to non-specific complaints of dyspepsia, she was also referred to the ultrasonography of the abdomen in March 2013. Surprisingly, multiple metastatic changes of the liver were revealed.

The results of basic laboratory parameters (blood count, haemocoagulation, and biochemistry) were within the normal range except for slightly increased GGT activity (1.18 μkat/l, normal values 0.08–0.65 μkat/l).

**Figure 1:**
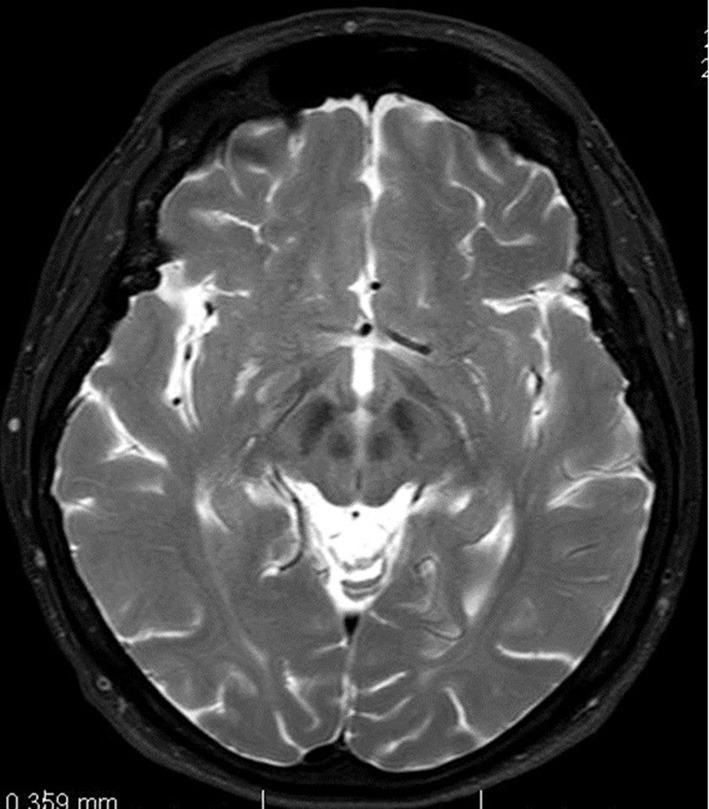
Magnetic resonance imaging of brain. T2-weighted axial scan. Changes in midbrain retrospectively evaluated as so-called face of giant panda sign.

In June 2013 the patient underwent a brain magnetic resonance imaging. A non-specific finding of multifocal encephalopathy supratentorially and bilaterally, with confluent changes in the brainstem region had been described (Figure 1).

**Figure 2:**
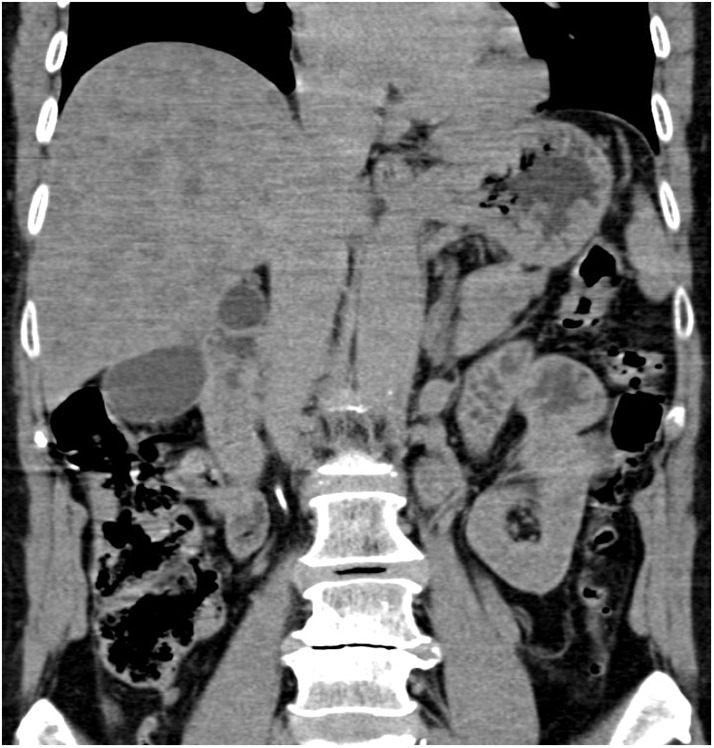
Abdominal computed tomography, non-enhanced coronary scan. Markedly inhomogenous liver with map-like hyperdense areas and multiple hypodense focal changes.

The CT revealed hepatomegaly with multiple focal changes in the liver (Figure 2).

A complete gynecological examination (including mammography), oesophagogastroduodenoscopy, colonoscopy, and a CT of the chest did not lead to the discovery of the potential origin of supposed metastases.

**Figure 3:**
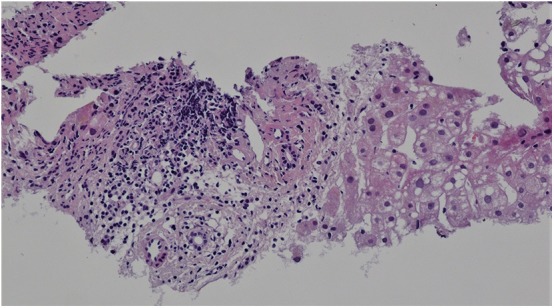
Liver histology. Hematoxylin-eosin staining, magnification x200. Portal space inflammatory cellularization composed of lymphocytes and plasmocytes. Severe macrovesicular steatosis.

Targeted biopsy of foci in the liver under CT control revealed severe diffuse steatofibrosis, with fibrously widened portobiliary spaces with multiplied biliary pseudoducts, inflammatory cellularization composed of lymphocytes and plasmocytes, some “piecemeal” necrosis of liver cells, severe macrovesicular steatosis, optically empty hepatocyte nuclei as a sign of an impaired glycide metabolism, some monocellular necrosis of liver cells with a small number of polymorphonuclear leukocytes and an image showing parenchymatous hepatocyte dystrophy. Accumulation of iron was not detected. Malignant structures were not detected (Figure 3).

Whole-body positron-emission tomography/computed tomography did not find hypermetabolic focal lesions in the liver or clear metastatic focal lesions at other locations.

Due to a finding of severe liver steatofibrosis of unclear etiology, the patient was subsequently, in November 2013, examined at the 1st Department of Internal Medicine. Neurological symptoms, namely combined resting-postural-kinetic coarse tremor of the head, chin and upper extremities, dominated in the objective finding. Biochemical blood tests revealed slightly increased activity of alaninaminotransferase (0.57 μkat/l, norm <0.55 μkat/l) and gamma glutamyl-transferase (1.06 μkat/l, norm <0.67 μkat/l). Blood count and haemocoagulation were within the normal range. Due to unclear hepatopathy and the present neurological symptoms, Wilson's disease was suspected, and then confirmed by the evaluation of copper metabolism parameters. Decreased concentration of serum ceruloplasmin (0.18 g/l, norm 0.20–0.60 g/L) and increased excretion of copper through urine (1752.60 nmol/24 h, norm <1,000 nmol/24 h) were detected. Eye examination with a slit lamp confirmed the presence of a Kayser-Fleischer ring. A genetic test revealed a mutation of the His1069Gln; 3207C>A gene for ATPase 7B in the homozygous state.

Chelation therapy with penicillamine was started, in a dose of 150 mg daily with a gradual increase (maximum daily dose of 1,050 mg) in combination with pyridoxine. The course of the therapy was complicated after 3 months by deterioration of her neurological symptoms attributed to an adverse effect of penicillamine. Due to very debilitating dystonic tremor of her head she was frequently injected with onabotulinumtoxin A into her posterior cervical muscles with substantial symptomatic effect.

**Figure 4:**
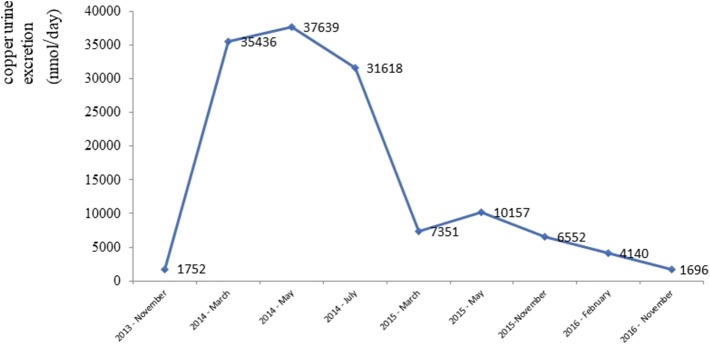
Urine copper excretion during chelating therapy.

After gradually lowering its dose (down to 450 mg daily) and commencing therapy with a zincum suphuricum in a dose of 200 mg daily, there was a gradual improvement in neurological symptoms. Copper urine excretion levels during the treatment depicts Figure 4.

## Discussion

Wilson's disease is a rare genetic disease that is treatable if it is discovered early—prior to the development of more severe damage, especially to the liver and brain.

If Wilson's disease is suspected, the first step involves examination of copper metabolism parameters (concentration of serum ceruloplasmin and excretion of copper through urine) and eye examination with a slit lamp with a focus on the Kayser-Fleischer ring. After that, genetic tests are done that confirm the diagnosis and enable targeted genetic tests as part of compulsory family screening in the patient's first-degree relatives. If the above tests do not unequivocally confirm Wilson's disease, while suspicion remains, histological examination of a liver sample with quantitative determination of the copper in dry matter is indicated (3).

Diagnosing the hepatic form of Wilson's disease can be complicated. Apart from a relatively wide spectrum of manifestation (ranging from an asymptomatic course with a laboratory finding of slightly increased aminotransferase activity to a fulminant hepatic failure) (4), the diagnostics can be complicated by frequently normal values of serum ceruloplasmin and the absence of a Kayser-Fleischer ring (5).

Diagnosing the neurological form of Wilson's disease is easier because almost all patients with this form have decreased serum ceruloplasmin values and a present Kayser-Fleischer ring (3).

The most common neurological symptoms of Wilson's disease include dysarthria, tremor (starting in upper extremities, and usually more dominant on one side), dystonia, dysdiadochokinesis, micrographia, rigidity, impaired posture and gait, hypomimia, characteristic open-mouth face expression, bradykinesia, uncontrollable fits of crying, swallowing disorders, salivation, and other signs of a damaged vegetative nervous system (4).

The unusual neurological symptoms of Wilson's disease comprise muscle cramps, undulating tongue movements, oculogyric crisis, and optic neuropathy and cough due to unconscious breathing muscle contractions (6).

Psychiatric presentation may occur at any stage of Wilson's disease. First symptoms may manifest in adolescence as worsening performance at school and behavioral changes. These symptoms may precede the hepatic and neurological symptomatology by a significant period of time. Other psychiatric manifestations include personality changes (antisocial behavior, irritability, and disinhibition), mood disorders (bipolar disorders, manic and hypomanic syndrome, depression, suicidal attempts), psychosis, anorexia, dyssomnias, sexual dysfunction, and cognitive impairment (7–9).

Other symptoms of Wilson's disease include hemolytic anemia (usually seen in manifestations with acute liver failure), kidney injury (in most cases tubular), osteoporosis, osteomalacia, arthralgia, arthritis, cholecystolitiasis, and endocrine disorders (amenorrhea and hyperthyroidism) (10).

In a large group of 1,223 patients, Wilson's disease manifested after 40 years of age in only 3.8% of cases, and 2/3 of these patients had neurological symptoms. In the aforementioned large group (11), Wilson's disease did not manifest after age 60 years. The available literature describes two cases of patients with a hepatic form (in one case with fulminant hepatic failure) and two cases with a mixed neurological and hepatic form of Wilson's disease (12–14), that manifested after age 60 years.

The factors influencing a phenotype (hepatic vs. neurological presentation, age of presentation) are not precisely known. The association between phenotype and genotype have not been confirmed. The important factor affecting phenotype is gender—in men Wilson's disease more commonly presents with neurological symptoms in older age. Hepatic presentation is more frequent in females (15). The other genetic, different environmental and epigenetic factors are considered to be important for clinical manifestation of the disease (16).

In the case of our patient (probably) due to her advanced age, the possibility of Wilson's disease was not considered, and the condition was erroneously assessed as part of her cerebral atherosclerosis and later as a sign of Parkinson's disease.

In patients with Wilson's disease, the brain MRI scan shows cerebral atrophy and symmetrical hyperintensity or mixed intensity in T2-weighted images changes in white matter of the cerebral hemispheres, basal ganglia, thalamus, cerebellum a brainstem.

Another well-known MRI findings that could help us to establish the diagnosis include the “face of giant panda sign” (mid-brain intensity changes on T2 weighted images—hyperintensity in the tegmentum, hypointensity of the superior colliculus and preservation of normal signal intensity in the red nuclei and lateral portion of the pars reticulata of the substantia nigra) as well as the “face of the miniature panda sign” or “trident sign,” or “panda cub sign” seen in the pontine tegmentum (intensity changes on T2 weighted image—relative hypointensity of the medial longitudinal fasciculi and central tegmetal tracts—the eyes of the panda, hyperintensity of the aqueduct opening into the fourth ventricle—nose and mouth of the panda, bounded inferiorly by the superior medullary velum) and “bright claustrum sign” (thin rim of T2 hyperintensity in lateral part of claustrum) (17–19).

The process that later indirectly led to making the correct diagnosis was triggered only by the ultrasonographic finding of a liver suspected of being affected by metastasis.

Ultrasonography of Wilson's disease patients shows a hyperechogenic liver as an imaging correlate of hepatic steatosis or fibrosis.

In the case of hepatic cirrhosis, the general ultrasonography signs that are found include: liver surface irregularities, thickened gall bladder walls, widened portal vein with decreased flow rate, portosystemic shunts, and splenomegaly. A value of the ratio of the size of the caudate lobe to the size of the right liver lobe equal to or >0.65 and a finding of a perihepatic hyperechogenic zone can point to Wilson's disease as a possible cause of liver cirrhosis (20, 21).

Parenchymatous heterogeneity with multiple hypo- or hyperechogenic nodules reminiscent of metastatic changes is a common finding during ultrasonography of the patients (22).

In our patient, it was the above finding reminiscent of metastatic liver damage that suggested a biopsy of the liver, which did not reveal malignant cells, and only a finding of steatofibrosis in the context of the above neurological symptoms led to suspecting an alternative diagnosis of Wilson's disease.

The suspected Wilson's disease was subsequently confirmed by a low concentration of serum ceruloplasmin, increased urine excretion of copper, the presence of a Kayser-Fleischer ring and genetic testing.

It is generally believed that Wilson disease is an illness of children or young adults. However, it is now becoming clear that this disease can present in a much older age group. Wilson disease should also be included in the differential diagnosis work-up of hepatic diseases and unclear neuropsychiatric syndromes in patients after age 60 years, especially because it is potentially treatable.

## Ethics Statement

Written informed consent was also obtained from the patient for the publication of this case report.

## Author Contributions

MŽ wrote the first draft of the manuscript. PVe analyzed liver histology and wrote section of manuscript. PB analyzed computed tomography and wrote section of manuscript. MŽ, MV, AT, and PVa contributed to the clinical management of the patient, manuscript revision, read, and approved the submitted version.

### Conflict of Interest

The authors declare that the research was conducted in the absence of any commercial or financial relationships that could be construed as a potential conflict of interest.
